# Reduced Interhemispheric White Matter Asymmetries in Medial Temporal Lobe Epilepsy With Hippocampal Sclerosis

**DOI:** 10.3389/fneur.2019.00394

**Published:** 2019-04-24

**Authors:** Xu Zhao, Zhi-qiang Zhou, Ying Xiong, Xu Chen, Ke Xu, Juan Li, Ying Hu, Xiao-long Peng, Wen-zhen Zhu

**Affiliations:** ^1^Department of Radiology, Tongji Hospital, Tongji Medical College, Huazhong University of Science and Technology, Wuhan, China; ^2^Department of Anesthesiology and Pain Medicine, Tongji Hospital, Tongji Medical College, Huazhong University of Science and Technology, Wuhan, China; ^3^Department of Neurosurgery, Tongji Hospital, Tongji Medical College, Huazhong University of Science and Technology, Wuhan, China; ^4^Department of Neurology, Tongji Hospital, Tongji Medical College, Huazhong University of Science and Technology, Wuhan, China

**Keywords:** medial temporal lobe epilepsy, hippocampal sclerosis, asymmetry, diffusion tensor imaging, tract-based spatial statistics

## Abstract

Mesial temporal lobe epilepsy (MTLE), one of the most common types of refractory focal epilepsy, has shown white matter abnormalities both within and beyond the temporal lobe. In particular, the white matter abnormalities in the ipsilateral hemisphere are more obvious than those in the contralateral hemisphere in MTLE, that is, the abnormalities present asymmetrical characteristics. However, very few studies have characterized the white matter microstructure asymmetry in MTLE patients specifically. Thus, we performed diffusion tensor imaging (DTI) to investigate the white matter microstructure asymmetries of patients with MTLE with unilateral hippocampal sclerosis (MTLE-HS). We enrolled 25 MTLE-HS (left MTLE-HS group, *n* = 13; right MTLE-HS group, *n* = 12) and 26 healthy controls (HC). DTI data were analyzed by tract-based spatial statistics (TBSS) to test the hemispheric differences across the entire white matter skeleton. We also conducted a two-sample paired *t*-test for 21 paired region of interests (ROIs) parceled on the basis of the ICBM-DTI-81 white-matter label atlas of bilateral hemispheres to test the hemispheric differences. An asymmetry index (AI) was calculated to further quantify the differences between the left and right paired-ROIs. It was found that the asymmetries of white matter skeletons were significantly lower in the MTLE-HS groups than in the HC group. In particular, the asymmetry traits were moderately reduced in the RMTLE-HS group and obviously reduced in the LMTLE-HS group. In addition, AI was significantly different in the RMTLE-HS group from the LMTLE-HS or HC group in the limbic system and superior longitudinal fasciculus (SLF). The current study found that the interhemispheric white matter asymmetries were significantly reduced in the MTLE-HS groups than in the HC group. The interhemispheric white matter asymmetries are distinctly affected in left and right MTLE-HS groups. The differences in AI among RMTLE-HS, LMTLE-HS, and HC involved the limbic system and SLF, which may have some pragmatic implications for the diagnosis of MTLE and differentiating LMTLE-HS from RMTLE-HS.

## Introduction

Both structural and functional asymmetries have long been observed in the human brain and thought to be a central principle of central nervous system architecture and to relate to differences in most cognitive and neurobehavioral functioning. The most consistent structural asymmetries are the rightward asymmetry of the frontal region and the leftward asymmetry of the occipital petalias and the planum temporale. The most striking functional lateralizations are the leftward asymmetry of verbal cognitive function and the rightward asymmetry of spatial cognitive function in most individuals (1–8). The alteration of the hemispheric asymmetries has been reported in some neurodevelopmental and psychiatric disorders, such as schizophrenia (9), prelingual deafness (10), and autism (11).

Mesial temporal lobe epilepsy (MTLE) is one of the most common types of refractory focal epilepsy, among which hippocampal sclerosis (HS) is the most frequent pathological change. About one-third of TLE cases are medically intractable, rendering them good candidates for surgical treatment. A key point for a successful surgery for TLE patients is to correctly recognize an epileptogenic focus, such as HS (12). Numerous studies have shown that the structural and/or functional changes of MTLE not only involve the temporal lobe but also extend beyond the temporal regions (13–16), and these structural and/or functional abnormalities present asymmetric characteristics. A review of the voxel-based morphometry (VBM) of TLE found significant volume reductions in 26 brain regions and a strong preference for asymmetric abnormalities to be observed ipsilateral to the epileptogenic zone (15). The white matter integrity of TLE assessed by diffusion tensor imaging (DTI) also shows more severe impairment in the ipsilateral than the contralateral hemisphere (17). The same asymmetric features of TLE were present in functional connectivity analysis as well (16). In particular, left MTLE (LMTLE) manifests as more widespread abnormalities and involves the contralateral hemisphere more widely than right MTLE (RMTLE) (13, 18–20). In addition, LMTLE often exhibits more intense cognitive impairment than RMTLE (21, 22). These data imply that LMTLE and RMTLE may be two different diseases with different pathogenesis (13, 23, 24).

Previous studies suggested that persistent seizure activities in TLE patients could produce white matter defects, such as reduced axonal density, axonal demyelination, and replacement of axons with glia (25, 26). DTI, as a noninvasive technique that can evaluate the integrity of the white matter microstructure *in vivo* based on measuring the restricted diffusion of water molecules in the brain, has been widely used in the analysis of white matter defects in TLE (27). Fractional anisotropy (FA) is a parameter of DTI that is used widely in describing the degree of anisotropy of a diffusion process and is thought to reflect fiber density, axonal diameter, and myelination in white matter. Mean diffusivity (MD) is another parameter of DTI that measures the extent of the diffusion of water molecules independent of directionality (28). Axial diffusivity (AD, the principal eigenvalue), also called the longitudinal diffusivity, reflects the diffusivity along the principal axis, and radial diffusivity (RD, the average of the 2 remaining eigenvalues) represents the diffusivity of the two minor axes. White matter anomalies have been found in TLE with using DTI, some of which are asymmetric (23, 29) and can be helpful in discriminating left TLE from right TLE (29). Moreover, recent advances have shown that the white matter asymmetry is probably the missing link between structural and functional lateralization (3, 30). Thus, we believe that asymmetric assessment of white matter in patients with MTLE may contribute to the understanding of its pathogenesis and may be useful in differentiating LMTLE from RMTLE.

However, the white matter asymmetric traits in MTLE are not fully understood. The aforementioned studies compared MTLE patients with controls and found inter-subject differences that could only provide indirect evidence of asymmetry. Although few studies focused on comparing the white matter of the two hemispheres directly, these analyses were restricted to specific fiber tracts and could not provide a comprehensive understanding of the asymmetric characteristics in white matter of MTLE. For example, Ahmadi et al. focused on eight pairs of whiter matter fiber tracts (29), and Kemmotsu et al. only investigated six pairs of fiber tracts that projected from the temporal lobe (23). Moreover, because the ROIs of fiber tracts were manually drawn, there was the possibility of intraoperator error (29). Tract-based spatial statistics (TBSS) is a whole-brain analytical method that requires no a priori assumptions and is widely used for the analysis of white matter microstructure (31). Therefore, in the current study, we examined the white matter asymmetry of DTI indices, including FA, MD, AD, and RD, by TBSS in patients with MTLE by contrasting the left and right hemispheres directly so that each individual served as his own control to diminish the variability between individuals. We also conducted a ROI-based quantitative analysis and compared the four DTI indices (FA, MD, AD, and RD) of 21 paired ROIs of bilateral hemispheres directly to add evidence for white matter asymmetries. The asymmetry index (AI) was compared among the LMTLE, RMTLE, and healthy control (HC) groups to distinguish LMTLE from RMTLE. As a homogeneous group of MTLE, we only included MTLE with unilateral HS (MTLE-HS) in the final statistical analysis to exclude any confounding factors. We hypothesized that the asymmetries of white matter microstructure in MTLE-HS would be different from HC and that AI would provide useful information for differentiating left MTLE-HS (LMTLE-HS) from right MTLE-HS (RMTLE-HS).

## Materials and Methods

### Subjects

We recruited 27 right-handed adult MTLE patients who were referred to Tongji hospital (Wuhan city, Hubei province, China) for the investigation of epilepsy. The diagnosis of MTLE was confirmed in all 27 patients according to the criteria defined by the Commission on Classification and Terminology of the International League Against Epilepsy (32). They were then divided into left MTLE or right MTLE based on clinical manifestations, video electroencephalography (v-EEG), neuroimaging results and/or PET-CT. To exclude the influence of different sides of epileptic foci on outcome, this study only included the homogeneous groups of MTLE patients with unilateral HS. Among these participants, 12 patients were pathologically confirmed to have HS by the subsequent surgical treatment, and the other 13 cases of HS were diagnosed according to the typical MRI findings (volume reduction of the ipsilateral hippocampus, disturbed internal structure, increased signal intensity on T2FLAIR, widening of ipsilateral temporal angle) (33). The exclusion criteria included a mismatch between seizure semiology, v-EEG and neuroimaging results; bilateral HS; lesions other than HS seen on MRI; and other neurologic disorders. One patient was excluded because of the discrepancy of v-EEG and MRI. Another 1 patient was excluded owing to excessive motion during the MRI, resulting in 25 patients with unilateral HS (LMTLE-HS group, *n* = 13; RMTLE-HS group, *n* = 12) whose imaging data were used in the final analysis. The age of first seizure and the epilepsy duration of these patients were also collected. Twenty-six right-handed healthy adults with matched age and gender were included as controls. There were no lesions on MRI and no history of neurologic or psychiatric illness in any controls. All 26 HCs were included in the final analysis. Consistency in age and gender among the RMTLE-HS, LMTLE-HS and HC groups was determined with 1-way ANOVA with Bonferroni corrected *post hoc* analysis and the χ2 test respectively. We conducted the Mann–Whitney *U* test for the age of first seizure and the epilepsy duration of RMTLE-HS and LMTLE-HS groups in SPSS 24, and the significance level was set at *p* < 0.05. All patients and controls included in this study gave written informed consent in accordance with the Declaration of Helsinki. This study was approved by the Ethical Committee of Tongji Hospital of Tongji Medical College of Huazhong University of Science and Technology.

### Image Acquisition

MR images were acquired using a 3.0-T MR scanner (Discovery MR750; GE Healthcare, Milwaukee, Wisconsin) with a 32-channel head coil. The subjects were padded with flexible foam to limit head motion. To exclude possible lesions specified in the exclusion criteria, all subjects underwent the standard structural brain scan, including axial T1WI, T2WI, T2FLAIR (TR/TE/TI 8400/160/2100 ms, section thickness 5 mm, section spacing 1.5 mm, matrix size 256 × 256, FOV 24.0 × 24.0 cm^2^, and NEX = 1). A sagittal three-dimensional T1-weighted-imaging brain volume (3D-T1BRAVO) (TR/TE/TI 8.2/3.2/450 ms, flip angle 12°, slice thickness 1 mm, sagittal slices 166, matrix size 256 × 256 × 160, FOV 25.6 × 25.6 cm^2^, and NEX = 1) and an oblique coronal T2FLAIR perpendicular to the long axis of the hippocampus were also acquired to observe the hippocampus and temporal lobe better. DTI data were obtained in the axial plane by using a single-shot diffusion-weighted echo-planar imaging pulse sequence with the following scanning protocol: TR/TE 8500/66.3 ms, FOV 25.6 × 25.6 cm^2^, matrix size 128 × 128, slice thickness 2 mm, number of slices 70, number of diffusion gradient directions 64, b = 0 and 1000 s/mm^2^, number of images at a b-value of 0 s/mm^2^ = 5, acceleration factor 2, and scan time 9 min 55 s.

### DTI Processing

The DTI data were preprocessing by the following steps: (1) Format transformation: MRIcron (http://www.nitrc.org/projects/mricron) was used to convert the image files in DICOM format into NIFTI format for further processing. (2) Eddy current correction: A FSL “eddy” tool (http://www.fmrib.ox.ac.uk/fsl/fslwiki/eddy) was used and all raw DTI images were aligned to the corresponding b0 images to correct eddy current distortions and motion artifacts. (3) Brain extraction: the nonbrain tissues such as scalp and skull were removed by using the Brain Extraction Tool (BET) (34). The robust brain center estimation was chosen and fractional intensity threshold was set as 0.2. (4) DTIFIT was used to calculate maps of FA, MD, AD, and RD for each subject.

We used TBSS (http://fsl.fmrib.ox.ac.uk/fsl/fslwiki/TBSS) to perform voxel-wise statistical analysis of the FA/MD/AD/RD images with the following steps (35): (1) Align all the FA images into the FMRIB58_FA_1 mm standard-space image through a nonlinear registration. (2) Write the nonlinear transform parameters to individual subjects and generate a mean FA skeleton from the actual subjects in our study, which represented the centers of all tracts common to the entire group. (3) Set an FA threshold of 0.2 to exclude nonskeleton voxels and generate individual skeletonized FA data. Each subject's MD, AD, RD images were then projected onto this skeleton. (4) Use the script “tbss_sym” to test the hemispheric differences. Firstly, the original asymmetric skeleton was dilated by one voxel. Next, the symmetric mean FA data was generated by flipping and averaging the original mean FA data, and then they were skeletonized to generate the initial symmetric skeleton. This skeleton was masked by the dilated original skeleton and was flipped and masked by the non-flipped image to ensure the skeleton was exactly symmetrical. Finally, a symmetrized skeleton was created for subsequent analysis. Each subject's aligned DTI data were projected onto this symmetrized skeleton. Asymmetry images were then created by the flipped minus original skeleton projected data, and the left side of the images were then zeroed. The resulting images were fed into voxelwise statistical analysis.

### Statistical Analysis

To test the asymmetry of FA/MD/AD/RD, we ran the permutation-based nonparametric 1-sample *t*-test (FSL Randomize tool with 5,000 permutations; http://fsl.fmrib.ox.ac.uk/fsl/fslwiki/randomise) twice separately (positive stands for left>right, negative stands for left<right). The significance level for *t*-tests (one-tailed) was set at *P* < 0.05, corrected for multiple comparisons by using Threshold-Free Cluster Enhancement (TFCE) with the familywise error (FWE) rate controlled. The rightward/leftward asymmetry on FA/MD/AD/RD was defined as having a larger/smaller FA/MD/AD/RD value of the right brain voxel/ROI than the left.

#### ROI-Based Quantitative Analysis

We used the ICBM-DTI-81 white-matter label atlas (36) as a standard to parcel the white matter into 48 ROIs. To test the hemisphere symmetry, 21 paired ROIs of bilateral hemispheres were included in the final analysis, excluding six ROIs in the midline involved both hemispheres. FA/MD/AD/RD was calculated by averaging the pixel values in these 21 paired ROIs for each subject separately. To eliminate potential confounders related to the innate asymmetry of human brain, we normalized all the DTI indices by using z scores based on the mean of the control group in a given hemisphere. We conducted the two-sample paired *t*-test for each paired ROI of each group by using SPSS, and the significance level was set at *P* < 0.05.

#### Asymmetry Index Calculation

An asymmetry index (AI) was calculated using the formula AI = 100 * (Right − Left)/[(Right + Left)/2] (11, 37) to further quantify the differences between the left and right paired ROIs mentioned above. A positive AI value indicated that the ROI value of the right hemisphere was greater than the corresponding left ROI, that is, rightward asymmetry, while a negative value stood for the opposite, that is, leftward asymmetry. For the statistical comparison of AIs among the three groups, a univariate ANOVA was used, and a *post hoc* analysis with Bonferroni correction was included for multiple comparisons and to determine the direction of AI differences among groups.

## Results

### Clinical Data

The clinical and demographic characteristics of the 3 subject groups are summarized in Table 1. No significant differences were found among the LMTLE-HS, RMTLE-HS, and HC in age (*P* = 0.531) or gender (*P* = 0.654). The age of first seizure and epilepsy duration did not show significant differences between the LMTLE-HS and RMTLE-HS groups (*P* = 0.611, *P* = 0.186, respectively) either.

**Table 1:** Demographic characteristics of the subjects.

	**LMTLE-HS** **(*n* = 13)**	**RMTLE-HS** **(*n* = 12)**	**HC** **(*n* = 26)**	**Significant differences** **(*P*-value)**
Age (mean ± SD years)	28.54 ± 9.84	31.25 ± 9.75	27.73 ± 8.08	0.531
Gender (male/female)	9/4	7/5	14/12	0.654
Median age at first seizure (years)	16	13	NA	0.611
Median epilepsy duration (years)	10	14	NA	0.186

*LMTLE-HS, left mesial temporal lobe epilepsy with hippocampal sclerosis; RMTLE-HS, right mesial temporal lobe epilepsy with hippocampal sclerosis; HC, healthy control; SD, standard deviation; NA, not applicable*.

### The TBSS Results of the Whole-Brain White Matter Asymmetry

**Figure 1:**
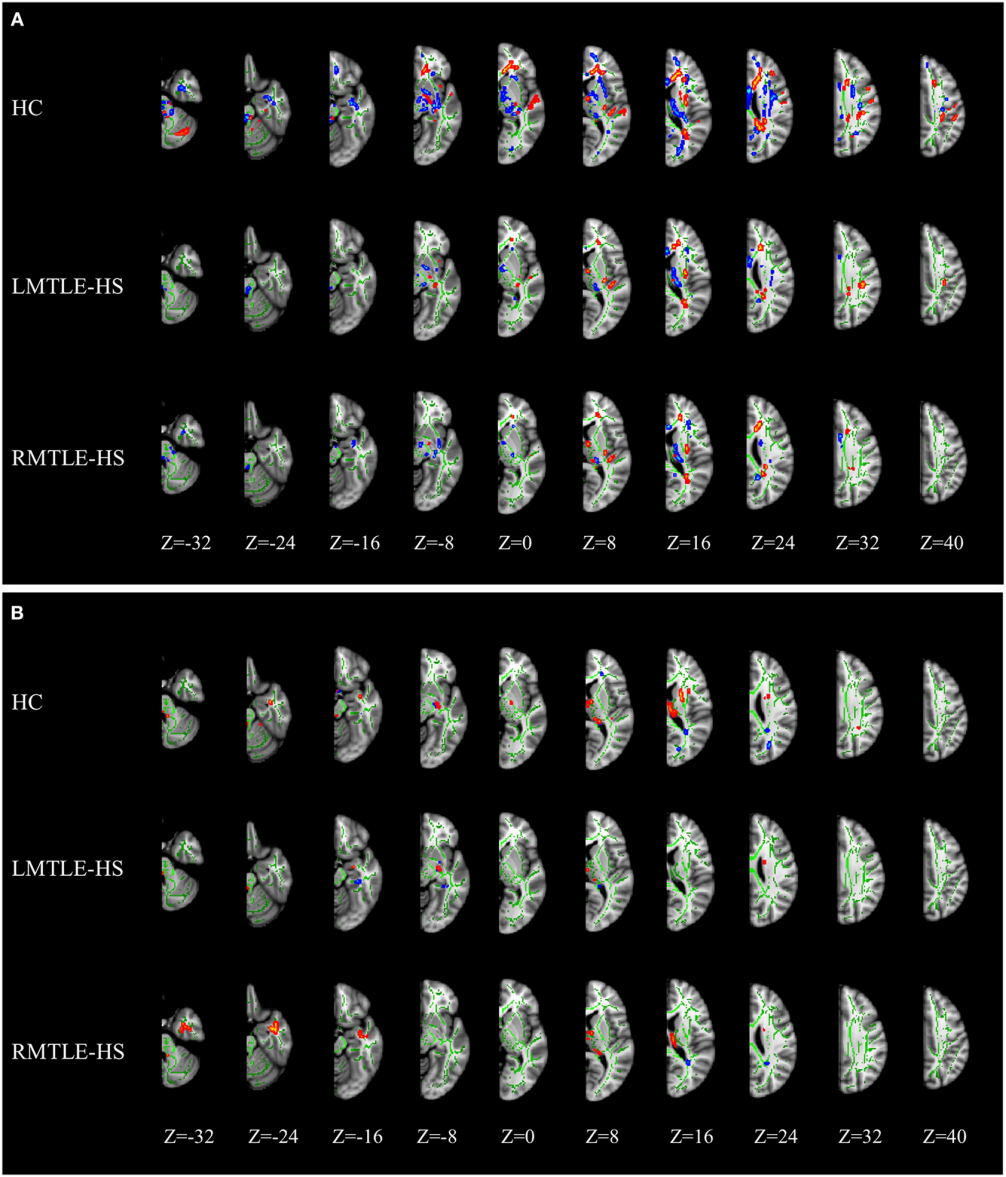
MRI images of significant tract-based spatial statistics (TBSS) clusters of white matter asymmetry in healthy controls, patients with left mesial temporal lobe epilepsy with hippocampal sclerosis (LMTLE-HS) and patients with right MTLE-HS. **(A)** Represents fractional anisotropy (FA) and **(B)** represents mean diffusivity (MD). Results are presented on the left hemisphere of the symmetric TBSS skeleton (depicted in green). Rightward asymmetry that was defined as having a larger FA/MD value of the right brain (R) than the left (L) (R > L) depicted in blue-light-blue and leftward asymmetry (L > R) in red-yellow.

As shown in Figure 1A, the TBSS skeleton analysis of asymmetry images showed a large proportion of leftward and rightward asymmetries across the whole brain of the HC group in FA, while both the LMTLE-HS and RMTLE-HS group displayed significantly reduced asymmetries than the HC group. The leftward asymmetry reductions in LMTLE-HS included the cingulum, superior corona radiata, external capsule, posterior limb of the internal capsule, and uncinate fasciculus (UF), and those in RMTLE-HS included the body of the corpus callosum, superior corona radiata, external capsule, and posterior limb of the internal capsule. The rightward asymmetry reductions in LMTLE-HS included the anterior corona radiate (ACR) and corpus callosum, and those in RMTLE-HS included the superior longitudinal fasciculus (SLF). In particular, the leftward asymmetry of the fornix (FORX) in FA seen in the HC group was reversed in the LMTLE-HS group. The MD map presented a relatively small area of asymmetry in the HC group compared to the FA map and a smaller area in the LMTLE-HS and RMTLE-HS group (Figure 1B). Asymmetric patterns of AD and RD were largely consistent with MD for the three groups (Figure S1).

### Results of ROI-Based Quantitative Analysis

The ROI-based quantitative analysis of FA also showed a large number of rightward and leftward asymmetries among the 21 paired ROIs (17 paired ROIs showed asymmetry features, among which nine showed rightward asymmetry and eight showed leftward asymmetry) in the HC group (Table S1). For MD, 16 paired ROIs presented asymmetric traits in the HC group, among which nine showed rightward asymmetry and seven showed leftward asymmetry (Table S2). AD and RD showed similar asymmetric features in the HC group as MD (Tables S3, S4).

**Figure 2:**
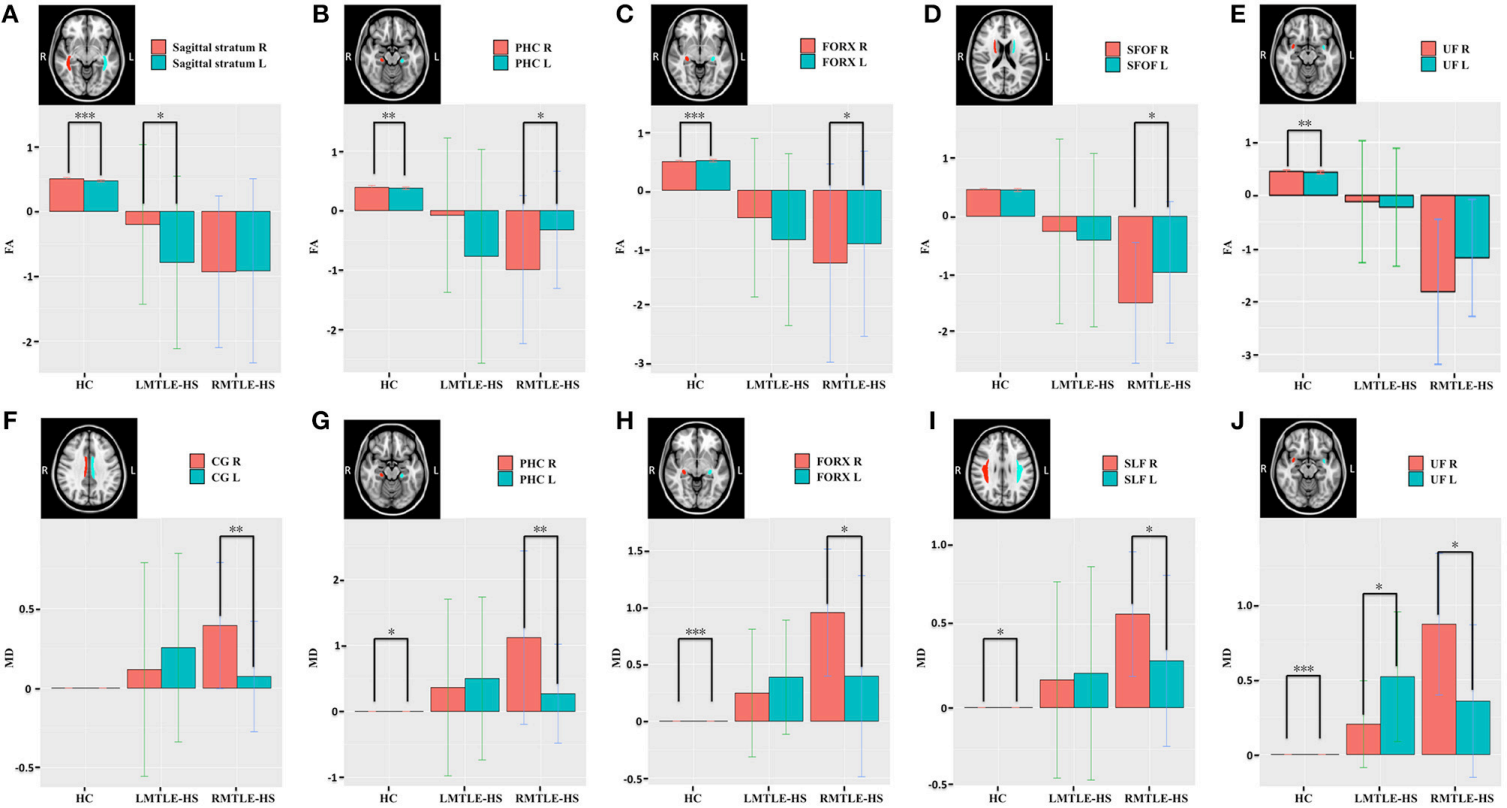
The paired-ROIs asymmetries in HC, LMTLE-HS, and RMTLE-HS were calculated by comparing the FA **(A–E)** or MD **(F–J)** values of the paired-ROIs in bilateral hemispheres. The FA and MD values of LMTLE-HS and RMTLE-HS were calculated by using z scores based on the mean of the HC in a given hemisphere. Red represents the FA or MD values of the right hemisphere and blue represents the FA or MD values of the left hemisphere. Rightward asymmetry was defined as having a larger FA/MD value of the right brain than the left, and leftward asymmetry was left ROI value larger than right (*P < 0.05; **P < 0.01; ***P < 0.001). PHC, parahippocampal cingulum; FORX, fornix; SFOF, superior fronto-occipital fasciculus; UF, uncinate fasciculus; CG, cingulum fibers within the cingulate gyrus; SLF, superior longitudinal fasciculus.

Compared to HC, the asymmetry traits were moderately reduced in the RMTLE-HS group and obviously reduced in the LMTLE-HS group. After eliminating the innate asymmetry of the human brain seen in the HC group, only eight paired ROIs [corticospinal tract (CST) (*P* = 0.02), medial lemniscus (*P* = 0.009), cerebral peduncle (*P* = 0.03), external capsule (*P* = 0.003), parahippocampal cingulum (PHC) (*P* = 0.01), FORX (*P* = 0.02), superior fronto-occipital fasciculus (SFOF) (*P* = 0.01), and tapetum (*P* = 0.04)] showed leftward asymmetry in FA, and 5 paired ROIs [cingulum (cingulate gyrus) (CG) (*P* = 0.004), PHC (*P* = 0.004), FORX (*P* = 0.01), SLF (*P* = 0.04) and UF (*P* = 0.01)] showed rightward asymmetry in MD in the RMTLE-HS group. Meanwhile, only two paired ROIs [posterior thalamic radiation (*P* = 0.01) and sagittal stratum (*P* = 0.01)] showed rightward asymmetry in FA and one paired ROI (UF) (*P* = 0.02) showed leftward asymmetry in MD in the LMTLE-HS group (Figure 2). Similarly, the results of ROI-based quantitative analysis of AD/RD were largely consistent with MD in both the LMTLE-HS and RMTLE-HS groups (Figure S2).

### Analysis of the Whole-Brain White Matter Asymmetry Index

**Figure 3:**
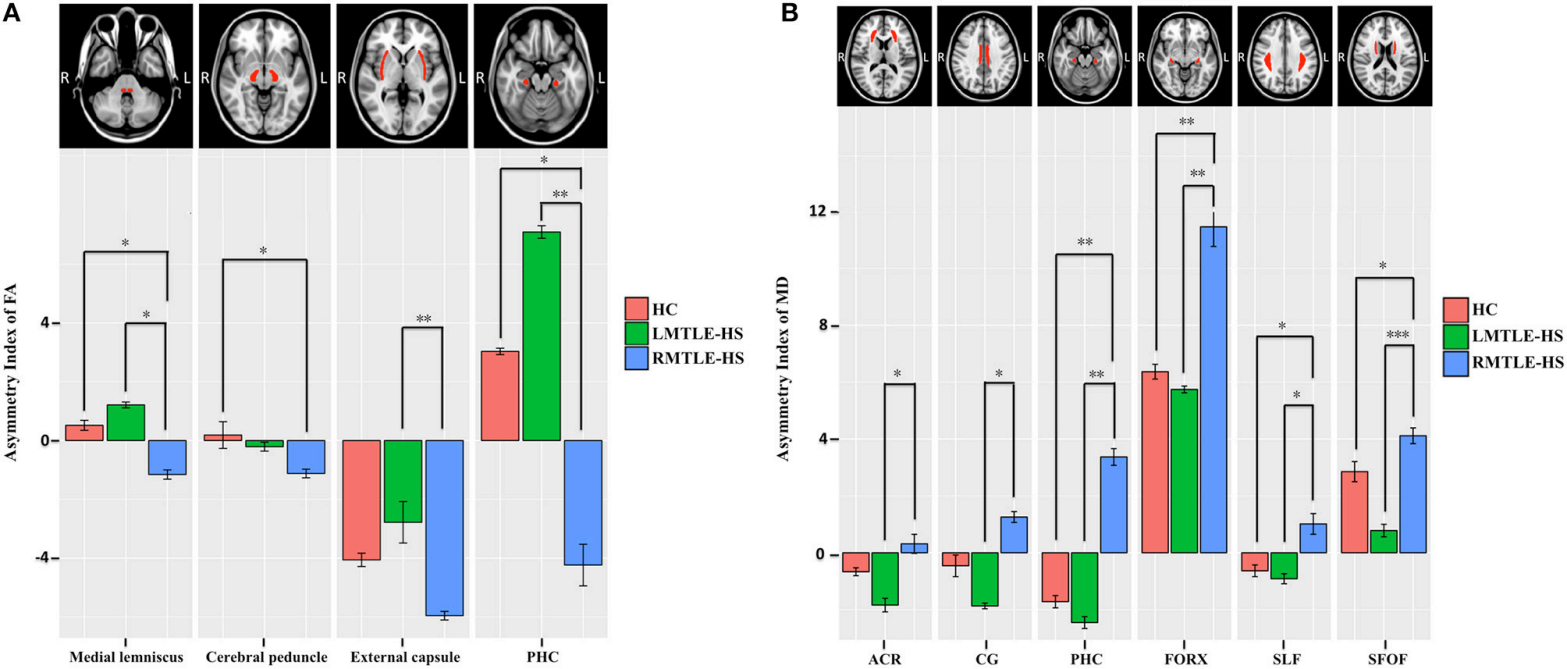
The asymmetry index (AI) differences of the paired-ROIs in bilateral hemispheres among HC, LMTLE-HS, and RMTLE-HS. **(A)** Represents the AI of FA and **(B)** represents the AI of MD. Red represents FA or MD values in HC; green, patients with LMTLE-HS; blue, patients with RMTLE-HS (*P < 0.05; **P < 0.01; ***P < 0.001). PHC, parahippocampal cingulum; ACR, anterior corona radiate; CG, cingulum fibers within the cingulate gyrus; FORX, fornix; SLF, superior longitudinal fasciculus; SFOF, superior fronto-occipital fasciculus.

The between-group analysis of AIs for the three groups showed no significant differences between HC and LMTLE-HS among all paired-ROIs. However, some differences were observed between HC and RMTLE-HS, such as PHC (*P* = 0.01) in FA (Figure 3A), and PHC (*P* = 0.002), FORX (*P* = 0.003), SLF (*P* = 0.02), and SFOF (*P* = 0.03) in MD (Figure 3B). The external capsule (*P* = 0.003) and PHC (*P* = 0.001) in FA (Figure 3A) and the ACR (*P* = 0.04), CG (*P* = 0.01), PHC (*P* = 0.002), FORX (*P* = 0.004), SLF (*P* = 0.01), and SFOF (*P* = 0.0001) in MD (Figure 3B) also showed significant differences between LMTLE-HS and RMTLE-HS. The AIs analysis in RD was similar to MD among the three groups (Figure S3B). In AD, FORX (*P* = 0.009) showed significant differences between HC and LMTLE-HS, and medial lemniscus (*P* = 0.01) and sagittal stratum (*P* = 0.03) showed significant differences between LMTLE-HS and RMTLE-HS (Figure S3A).

## Discussion

The structural asymmetry of white matter microstructure in patients with MTLE-HS has not yet been comprehensively investigated. In this study, we used DTI to investigate the hemispheric asymmetry of the white matter microstructure in patients with MTLE-HS. Our findings showed that interhemispheric asymmetries were significantly lower in the MTLE-HS groups than in the HC group. In particular, the asymmetric areas were smaller in the LMTLE-HS than the RMTLE-HS group. In addition, AI was significantly different in the RMTLE-HS group from that in the LMTLE-HS or HC group in the limbic system and SLF. Taken together, our results indicate that reductions of asymmetry of the white matter microstructure in patients with MTLE-HS compared to HC and the asymmetric alterations might be a useful clue in understanding the lateralization of seizure foci.

### Brain Asymmetry in Patients With MTLE

MTLE-HS is one of the most common types of refractory focal epilepsy. Although many studies have shown that both structural and functional abnormalities were asymmetric in MTLE patients (13, 16, 19, 22, 23, 29), these findings have not been found consistently. Some studies have reported that the alterations in the ipsilateral hemisphere were more severe than those in the contralateral hemisphere in MTLE (15, 17). In particular, the majority of these studies showed that the LMTLE patients presented more diffused and bilateral abnormalities than the RMTLE patients (13, 16, 18–20, 23, 29). In contrast, some other studies reported the opposite results (24, 38) that alterations in the RMTLE group were more severe than in the LMTLE group. However, very few studies have focused specifically on patients with MTLE to characterize the asymmetry. The different asymmetric patterns between LMTLE and RMTLE remain unclear, and the importance of these asymmetries for understanding MTLE should be noted. First, the abnormal asymmetry of MTLE is associated with lateralization of seizure. For example, M.E. Ahmadi et al. found that using the asymmetric features of some fiber tracts could increase the lateralization of seizure foci to 90% of all cases (29). Concha et al. also found that tract segments correctly lateralized 87% of patients (39). Second, abnormal asymmetry of MTLE might be associated with the cognitive dysfunction in MTLE. For example, previous studies reported that the LMTLE-HS group showed worse performance on many neuropsychological tests (including verbal memory, general memory, and delayed recall) than the RMTLE-HS group (16). Therefore, understanding the underlying mechanisms of the differences in asymmetry between MTLE and HCs may play an important role in the clinic.

### Asymmetry Abnormalities of White Matter Microstructure in MTLE-HS

TBSS analysis showed significant reductions of the asymmetries of whole-brain white matter in FA in the MTLE-HS groups compared to the HC group. These findings indicate that the interhemispheric asymmetry of patients with MTLE-HS is impaired (11). Structural asymmetry is a characteristic of human brain organization and is beneficial for the functional specializations of the two hemispheres, in particular cognitive processes (1, 5, 7, 40). Gotts et al. provides direct evidence that the strength of functional lateralization is correlated with the level of cognitive ability in humans (41). Furthermore, aberrant interhemispheric asymmetries are associated with some neuropathologies, such as prelingual deafness, autism, schizophrenia, and epilepsy (9–11, 42). Therefore, we assumed that the reduction of white matter asymmetry of MTLE-HS might be related to the reported cognitive dysfunctions (16).

Some previous studies indicated that the innate side-to-side cerebral asymmetry could contribute to the observed difference between LTLE and RTLE (43). However, whether the asymmetric changes of MTLE patients are associated with the innate human brain asymmetry needs further analysis. Here, to eliminate the probable influences of innate human brain asymmetry, we normalized all the DTI indices by using z scores based on the mean of the control group in a given hemisphere. Specifically, after eliminating the innate asymmetry of the human brain seen in the HC group, we found only rightward asymmetry in FA and only leftward asymmetry in MD in the LMTLE-HS group and only leftward asymmetry in FA and only rightward asymmetry in MD in the RMTLE-HS group. The AD and RD showed similar asymmetric features as MD. Our findings support the previous results that more pronounced changes ipsilateral to the seizure focus were presented in LMTLE/RMTLE by using whole-brain analysis and by comparing the bilateral hemispheres directly (17, 23, 29). The FA reduction and MD increase in the ipsilateral hemisphere to the seizure focus of MTLE have been consistently reported. We speculated that RMTLE-HS/LMTLE-HS influenced the ipsilateral side of these paired ROIs more severely, resulting in reduced/elevated FA/MD values of the ipsilateral side and thus manifesting as asymmetric features.

### The Different White Matter Asymmetrical Patterns Between LMTLE-HS and RMTLE-HS

The asymmetry alterations appeared differently between LMTLE-HS and RMTLE-HS. The asymmetric ROIs were smaller in the LMTLE-HS group than in the RMTLE-HS group. A possible explanation for this finding is that LMTLE-HS affected the bilateral hemispheres almost equally and thus the innate asymmetry of the human brain was nearly not affected by LMTLE-HS. The reliability of these findings is further supported by the ROI result that the AI between LMTLE-HS and HC did not present significant differences. Our findings were partially consistent with many previous studies. Abnormalities in LMTLE-HS are more widespread and bilateral than those in RMTLE-HS (13, 20, 23). For example, Kemmotsu et al. reported that patients with LTLE showed smaller FA values in the ipsilateral hemisphere of the UF and ILF, as well as a similar trend in the PHC, ARC and IFOF. RTLE patients showed a trend of smaller ipsilateral FA values only in the PHC (23). Recently, a large-scale brain network study also showed that LMTLE-HS had a more intricate bihemispheric dysfunction than RMTLE-HS (13). For RMTLE-HS, the impairment was relatively more confined to the ipsilateral hemisphere, and therefore, the innate asymmetry of the human brain was changed. The exact underlying mechanisms remain to be elucidated. They might have some relationship with the greater vulnerability of the left hemisphere to early insults and the wider propagation of left hemisphere seizures (23). It is also possible that LMTLE and RMTLE are etiologically distinct and pathologically different syndromes from the outset (29).

### The Different Asymmetry Alterations of the Limbic System and SLF Among the Three Groups

Some differences were observed in AI analysis among the three groups, such as in the CG, PHC, and FORX. It should be noted that the CG, PHC, and FORX are parts of the limbic system. Within this system, the FORX serves as the internal ring that projects from the hippocampus to the mammillary bodies, while the cingulum serves as the external ring interconnecting the entorhinal cortex and the cingulate gyrus (44, 45). It has long been proposed that TLE may be a probable manifestation of limbic system dysfunction (46), and the relevance of the limbic system as a MTLE-HS neural circuit has been well established (47, 48). For example, Despina Liacu et al. found that the bilateral cingulum and FORX in MTLE-HS groups showed significant DTI index abnormalities compared with controls (47). H. Urbach also found a bilateral reduction of the streamline counts of cingulum association fibers projecting from the cingulate gyrus to the parahippocampal gyrus in MTLE-HS (48). Thus, the differences in AI of these components of the limbic system among HC, RMTLE-HS, and LMTLE-HS may have some pragmatic implications as to the diagnosis MTLE and the differentiation of LMTLE-HS from RMTLE-HS.

Furthermore, the AI of SLF in MD also showed significant differences among HC, RMTLE-HS and LMTLE-HS. SLF is a bilateral white matter association fiber tract connecting the frontal, occipital, parietal, and temporal lobes (49). SLF plays an important role in many complex cognitive processes, including working memory, and language processing (50, 51). The abnormalities of SLF in TLE have been found in many studies (23, 29), and the association between SLF and memory and language impairments have also been presented in TLE (52). For example, the arcuate fasciculus (AF), a part of the SLF of TLE, has been associated with poorer verbal memory and naming performance. Regression analyses revealed that AF independently predicted cognitive performance after controlling for hippocampal volume (52). Thus, the different AIs of the SLF among the three groups in our study might indicate the different cognitive functions associated with SLF, but this inference needs further verification because of the lack of cognitive function evaluation in the current study.

## Limitation

Some limitations of the current study should be mentioned. First, we did not evaluate the subjects' cognitive functions and thus could not clarify the relationship between asymmetry and cognitive functions. Such cognitive functions as language have been observed to be associated with brain asymmetry (5). We would include cognitive evaluation in future studies. Second, we only analyzed the white matter asymmetry of MTLE patients. Further research about the functional and other structural asymmetries should be included in future studies, which might provide a comprehensive understanding of the asymmetry characteristics of MTLE.

## Conclusion

The current study found that the interhemispheric asymmetries in FA were significantly lower in both the LMTLE-HS group and the RMTLE-HS group than in the HC group. The ROI-based quantitative analysis provided further evidence for the previous result that RMTLE-HS/LMTLE-HS influences the ipsilateral hemisphere more severely and that impairment in LMTLE-HS may be more diffuse and bilateral than that in RMTLE-HS. The differences in AI among RMTLE-HS, LMTLE-HS, and HC involved the limbic system and the SLF, which may have some pragmatic implications as to the diagnosis of MTLE and the differentiation of LMTLE-HS from RMTLE-HS.

## Ethics Statement

This study was carried out in accordance with the recommendations of the Ethical Committee of Tongji Hospital of Tongji Medical College of Huazhong University of Science and Technology with written informed consent from all subjects. All subjects gave written informed consent in accordance with the Declaration of Helsinki. The protocol was approved by the Ethical Committee of Tongji Hospital of Tongji Medical College of Huazhong University of Science and Technology.

## Author Contributions

XZ, ZZ, and WZ were responsible for the study design, data interpretation and writing the manuscript. YX, XZ, XP, and ZZ contributed to MRI data acquisition, TBSS processing, ROI-based quantitative analyzing and AI analyzing. XC, KX, JL, and YH assisted in recruiting subjects, acquiring MRI data and collecting clinical data. WZ revised the first draft of the manuscript. All authors critically reviewed and approved the submitted version.

### Conflict of Interest Statement

The authors declare that the research was conducted in the absence of any commercial or financial relationships that could be construed as a potential conflict of interest.
