# Supplementary material for: Reduced Interhemispheric White Matter Asymmetries in Medial Temporal Lobe Epilepsy With Hippocampal Sclerosis
Source: Front Neurol. 2019 Apr 24;10:394. doi: 10.3389/fneur.2019.00394 (PMC6491759; doi:10.3389/fneur.2019.00394)
Supplement: Supplementary file 1 [file Data_Sheet_1.docx]

Supplementary Material

**Reduced interhemispheric white matter asymmetries in medial temporal lobe epilepsy with hippocampal sclerosis**

**Xu Zhao^1^, Zhi-qiang Zhou^2^, Ying Xiong^1^, Xu Chen^3^, Ke Xu^4^, Juan Li^1^, Ying Hu^1^, Xiao-long Peng^1^, Wen-zhen Zhu^1＊^**

^1^Department of Radiology, Tongji Hospital, Tongji Medical College, Huazhong University of Science and Technology, Wuhan, China

^2^Department of Anesthesiology and Pain Medicine, Tongji Hospital, Tongji Medical College, Huazhong University of Science and Technology, Wuhan, China

^3^Department of Neurosurgery, Tongji Hospital, Tongji Medical College, Huazhong University of Science and Technology, Wuhan, China

^4^Department of Neurology, Tongji Hospital, Tongji Medical College, Huazhong University of Science and Technology, Wuhan, China

***Correspondence:**

Wen-zhen Zhu

zhuwenzhen8612@163.com

**1** **Supplementary Tables**

**TABLE S1.**  Quantitative analysis for FA values of each paired ROI of each group

| FA of ROIs | HC | | LMTLE-HS | | RMTLE-HS | |
| --- | --- | --- | --- | --- | --- | --- |
|  | t | *P* value | t | *P* value | t | *P* value |
| corticospinal tract | **-2.22** | **0.04*** | -0.18 | 0.86 | **-2.52** | **0.02*** |
| medial lemniscus | 1.31 | 0.20 | 1.34 | 0.20 | **-1.32** | **0.009**** |
| inferior cerebellar peduncle | 1.42 | 0.17 | 0.50 | 0.63 | -1.19 | 0.26 |
| superior cerebellar peduncle | **16.88** | **0.00***** | 1.12 | 0.29 | 1.46 | 0.17 |
| cerebral peduncle | 0.81 | 0.42 | -0.75 | 0.47 | **-2.39** | **0.03*** |
| anterior limb of internal capsule | **-3.61** | **0.001**** | 0.51 | 0.62 | -1.005 | 0.34 |
| posterior limb of internal capsule | **-5.11** | **0.00***** | 0.13 | 0.90 | -0.563 | 0.59 |
| retrolenticular part of internal capsule | **-3.63** | **0.001**** | 1.22 | 0.25 | 0.85 | 0.41 |
| anterior corona radiata | **2.93** | **0.007**** | -1.74 | 0.11 | -1.41 | 0.19 |
| superior corona radiata | **-4.19** | **0.00***** | 0.23 | 0.82 | 1.01 | 0.33 |
| posterior corona radiata | **8.92** | **0.00***** | 1.64 | 0.13 | 2.07 | 0.06 |
| posterior thalamic radiation | **3.48** | **0.002**** | **2.75** | **0.01*** | 0.89 | 0.40 |
| sagittal stratum | **11.59** | **0.00***** | **2.96** | **0.01*** | -0.07 | 0.95 |
| external capsule | **-10.80** | **0.00***** | 1.45 | 0.17 | **-3.83** | **0.003**** |
| cingulum （cingulate gyrus） | **-13.65** | **0.00***** | 0.02 | 0.98 | -0.34 | 0.74 |
| parahippocampal cingulum | **2.98** | **0.006**** | 1.93 | 0.08 | **-3.09** | **0.01*** |
| fornix | **-4.92** | **0.00***** | 1.35 | 0.20 | **-2.62** | **0.02*** |
| superior longitudinal fasciculus | **3.31** | **0.003**** | 0.87 | 0.40 | -1.03 | 0.33 |
| superior fronto-occipital fasciculus | 1.67 | 0.11 | 0.47 | 0.65 | **-2.75** | **0.01*** |
| uncinate fasciculus | **3.27** | **0.003**** | 0.50 | 0.62 | -2.07 | 0.06 |
| tapetum | **20.51** | **0.00***** | -0.91 | 0.38 | **-2.31** | **0.04*** |

FA, fractional anisotropy; ROI, region of interest; LMTLE-HS, left mesial temporal lobe epilepsy with hippocampal sclerosis; RMTLE-HS, right mesial temporal lobe epilepsy with hippocampal sclerosis; HC, healthy control. T>0 represents rightward asymmetry and t<0 represents leftward asymmetry. The FA values of LMTLE-HS and RMTLE-HS were calculated by using z scores based on the mean of the HC in a given hemisphere. *P* and t values were calculated using the two-sample paired t-test model (**P*<0.05; ***P*<0.01; ****P*<0.001).

**TABLE S2.** Quantitative analysis for MD values of each paired ROI of each group

| MD of ROIs | HC | | LMTLE-HS | | RMTLE-HS | |  |  |
| --- | --- | --- | --- | --- | --- | --- | --- | --- |
|  | t | *P value* | t | *P value* | t | *P value* | |  |
| corticospinal tract | 0.52 | 0.61 | -1.84 | 0.09 | 2.00 | 0.07 | |  |
| medial lemniscus | -1.77 | 0.09 | 1.36 | 0.20 | -0.85 | 0.42 | |  |
| inferior cerebellar peduncle | **2.61** | **0.02*** | -1.70 | 0.12 | -1.04 | 0.32 | |  |
| superior cerebellar peduncle | **-22.94** | **0.00***** | 0.74 | 0.48 | -0.81 | 0.43 | |  |
| cerebral peduncle | **6.57** | **0.00***** | 1.60 | 0.14 | 1.47 | 0.17 | |  |
| anterior limb of internal capsule | **12.25** | **0.00***** | 0.02 | 0.99 | 0.13 | 0.90 | |  |
| posterior limb of internal capsule | **13.26** | **0.00***** | -0.45 | 0.66 | 0.15 | 0.88 | |  |
| retrolenticular part of internal capsule | 1.16 | 0.26 | -1.16 | 0.27 | 0.99 | 0.34 | |  |
| anterior corona radiata | -1.99 | 0.06 | -1.72 | 0.11 | 1.16 | 0.27 | |  |
| superior corona radiata | **6.49** | **0.00***** | -1.27 | 0.23 | 0.007 | 0.10 | |  |
| posterior corona radiata | **-6.28** | **0.00***** | -1.09 | 0.30 | -0.01 | 0.99 | |  |
| posterior thalamic radiation | **-6.95** | **0.00***** | 0.06 | 0.96 | 0.08 | 0.94 | |  |
| sagittal stratum | **-7.28** | **0.00***** | -1.26 | 0.23 | 1.83 | 0.09 | |  |
| external capsule | **8.00** | **0.00***** | -0.45 | 0.66 | 1.62 | 0.13 | |  |
| cingulum （cingulate gyrus） | -1.30 | 0.21 | -1.39 | 0.19 | **3.60** | **0.004**** | |  |
| parahippocampal cingulum | **-2.10** | **0.04*** | -0.87 | 0.40 | **3.65** | **0.004**** | | |
| fornix | **6.91** | **0.00***** | -1.28 | 0.22 | **3.07** | **0.01*** | |  |
| superior longitudinal fasciculus | **-2.76** | **0.01*** | -0.64 | 0.53 | **2.31** | **0.04*** | |  |
| superior fronto-occipital fasciculus | **6.14** | **0.00***** | -1.00 | 0.34 | 1.31 | 0.22 | |  |
| uncinate fasciculus | **6.39** | **0.00***** | **-2.61** | **0.02*** | **2.98** | **0.01*** | |  |
| tapetum | **-16.82** | **0.00***** | -1.53 | 0.15 | -0.18 | 0.86 | |  |

MD, mean diffusivity; ROI, region of interest; LMTLE-HS, left mesial temporal lobe epilepsy with hippocampal sclerosis; RMTLE-HS, right mesial temporal lobe epilepsy with hippocampal sclerosis; HC, healthy control. T>0 represents rightward asymmetry and t<0 represents leftward asymmetry. The MD values of LMTLE-HS and RMTLE-HS were calculated by using z scores based on the mean of the HC in a given hemisphere. *P* and t values were calculated using the two-sample paired t-test model (**P*<0.05; ***P*<0.01; ****P*<0.001).

**TABLE S3.** Quantitative analysis for AD values of each paired ROI of each group

| AD of ROIs | HC | | LMTLE-HS | | RMTLE-HS | |  |  |
| --- | --- | --- | --- | --- | --- | --- | --- | --- |
|  | t | *P value* | t | *P value* | t | *P value* | |  |
| corticospinal tract | 0.09 | 0.93 | -1.81 | 0.10 | 0.27 | 0.79 | |  |
| medial lemniscus | -0.55 | 0.59 | 1.93 | 0.08 | **-2.54** | **0.03*** | |  |
| inferior cerebellar peduncle | **2.56** | **0.02*** | 0.09 | 0.93 | -1.02 | 0.33 | |  |
| superior cerebellar peduncle | **-15.70** | **0.00***** | 0.78 | 0.45 | -1.10 | 0.29 | |  |
| cerebral peduncle | **6.37** | **0.00***** | 1.75 | 0.11 | 0.02 | 0.98 | |  |
| anterior limb of internal capsule | **10.30** | **0.00***** | -0.02 | 0.98 | -0.13 | 0.90 | |  |
| posterior limb of internal capsule | **15.03** | **0.00***** | -0.43 | 0.67 | -0.11 | 0.92 | |  |
| retrolenticular part of internal capsule | **-2.19** | **0.04*** | 0.50 | 0.62 | **2.35** | **0.04*** | |  |
| anterior corona radiata | 0.95 | 0.35 | -1.68 | 0.12 | 0.73 | 0.48 | |  |
| superior corona radiata | **3.47** | **0.002**** | -0.42 | 0.69 | 0.58 | 0.58 | |  |
| posterior corona radiata | -0.51 | 0.62 | 0.32 | 0.75 | 0.95 | 0.36 | |  |
| posterior thalamic radiation | **-5.78** | **0.00***** | -0.25 | 0.81 | 0.13 | 0.90 | |  |
| sagittal stratum | **-2.78** | **0.01*** | -1.85 | 0.09 | **2.35** | **0.04*** | |  |
| external capsule | **4.07** | **0.00***** | 0.18 | 0.86 | 1.51 | 0.16 | |  |
| cingulum （cingulate gyrus） | **-9.38** | **0.00***** | -0.83 | 0.42 | **3.02** | **0.01*** | |  |
| parahippocampal cingulum | -0.35 | 0.73 | 0.43 | 0.68 | **2.79** | **0.02*** | | |
| fornix | **5.98** | **0.00***** | 1.17 | 0.27 | **2.47** | **0.03*** | |  |
| superior longitudinal fasciculus | 0.59 | 0.56 | 0.27 | 0.80 | 1.44 | 0.18 | |  |
| superior fronto-occipital fasciculus | **6.47** | **0.00***** | -0.64 | 0.54 | 0.78 | 0.45 | |  |
| uncinate fasciculus | **7.39** | **0.00***** | -2.05 | 0.06 | 0.53 | 0.60 | |  |
| tapetum | **-12.1** | **0.00***** | -1.79 | 0.10 | -0.90 | 0.39 | |  |

AD, axial diffusivity; ROI, region of interest; LMTLE-HS, left mesial temporal lobe epilepsy with hippocampal sclerosis; RMTLE-HS, right mesial temporal lobe epilepsy with hippocampal sclerosis; HC, healthy control. t>0 represents rightward asymmetry and t<0 represents leftward asymmetry. The AD values of LMTLE-HS and RMTLE-HS were calculated by using z scores based on the mean of the HC in a given hemisphere. *P* and t values were calculated using the two-sample paired t-test model (**P*<0.05; ***P*<0.01; ****P*<0.001).

**TABLE S4.** Quantitative analysis for RD values of each paired ROI of each group

| RD of ROIs | HC | | LMTLE-HS | | RMTLE-HS | |  |  |
| --- | --- | --- | --- | --- | --- | --- | --- | --- |
|  | t | *P value* | t | *P value* | t | *P value* | |  |
| corticospinal tract | 0.95 | 0.35 | -1.60 | 0.14 | 1.86 | 0.09 | |  |
| medial lemniscus | -2.11 | 0.05 | 0.10 | 0.92 | 1.37 | 0.20 | |  |
| inferior cerebellar peduncle | **2.53** | **0.02*** | -2.02 | 0.07 | -1.65 | 0.13 | |  |
| superior cerebellar peduncle | **-25.64** | **0.00***** | 0.36 | 0.72 | -0.43 | 0.68 | |  |
| cerebral peduncle | **5.63** | **0.00***** | 1.46 | 0.17 | 1.92 | 0.08 | |  |
| anterior limb of internal capsule | **11.81** | **0.00***** | -0.26 | 0.80 | -0.12 | 0.91 | |  |
| posterior limb of internal capsule | **10.40** | **0.00***** | -0.32 | 0.76 | 0.27 | 0.79 | |  |
| retrolenticular part of internal capsule | **5.36** | **0.00***** | -1.61 | 0.13 | -0.42 | 0.68 | |  |
| anterior corona radiata | **-3.72** | **0.00***** | -0.99 | 0.34 | 1.06 | 0.31 | |  |
| superior corona radiata | **5.09** | **0.00***** | -1.11 | 0.29 | -0.44 | 0.67 | |  |
| posterior corona radiata | **-9.73** | **0.00***** | -1.34 | 0.20 | -0.55 | 0.59 | |  |
| posterior thalamic radiation | **-7.35** | **0.00***** | -1.26 | 0.23 | -0.12 | 0.91 | |  |
| sagittal stratum | **-9.77** | **0.00***** | -1.22 | 0.25 | **2.33** | **0.04*** | |  |
| external capsule | **9.71** | **0.00***** | -0.92 | 0.38 | 1.27 | 0.23 | |  |
| cingulum （cingulate gyrus） | **7.20** | **0.00***** | -1.47 | 0.17 | **3.10** | **0.01*** | |  |
| parahippocampal cingulum | **-2.56** | **0.02*** | -1.83 | 0.09 | **3.57** | **0.00***** | | |
| fornix | **6.73** | **0.00***** | -1.74 | 0.11 | **3.43** | **0.01*** | |  |
| superior longitudinal fasciculus | **-3.58** | **0.00***** | -0.38 | 0.71 | 2.15 | 0.06 | |  |
| superior fronto-occipital fasciculus | **3.99** | **0.00***** | -0.99 | 0.34 | 1.73 | 0.11 | |  |
| uncinate fasciculus | **2.38** | **0.03*** | -2.23 | 0.05 | **3.19** | **0.01*** | |  |
| tapetum | **-19.00** | **0.00***** | -1.32 | 0.21 | 0.20 | 0.85 | |  |

RD, radial diffusivity; ROI, region of interest; LMTLE-HS, left mesial temporal lobe epilepsy with hippocampal sclerosis; RMTLE-HS, right mesial temporal lobe epilepsy with hippocampal sclerosis; HC, healthy control. t>0 represents rightward asymmetry and t<0 represents leftward asymmetry. The RD values of LMTLE-HS and RMTLE-HS were calculated by using z scores based on the mean of the HC in a given hemisphere. *P* and t values were calculated using the two-sample paired t-test model (**P*<0.05; ***P*<0.01; ****P*<0.001).

**2** **Supplementary Figures**


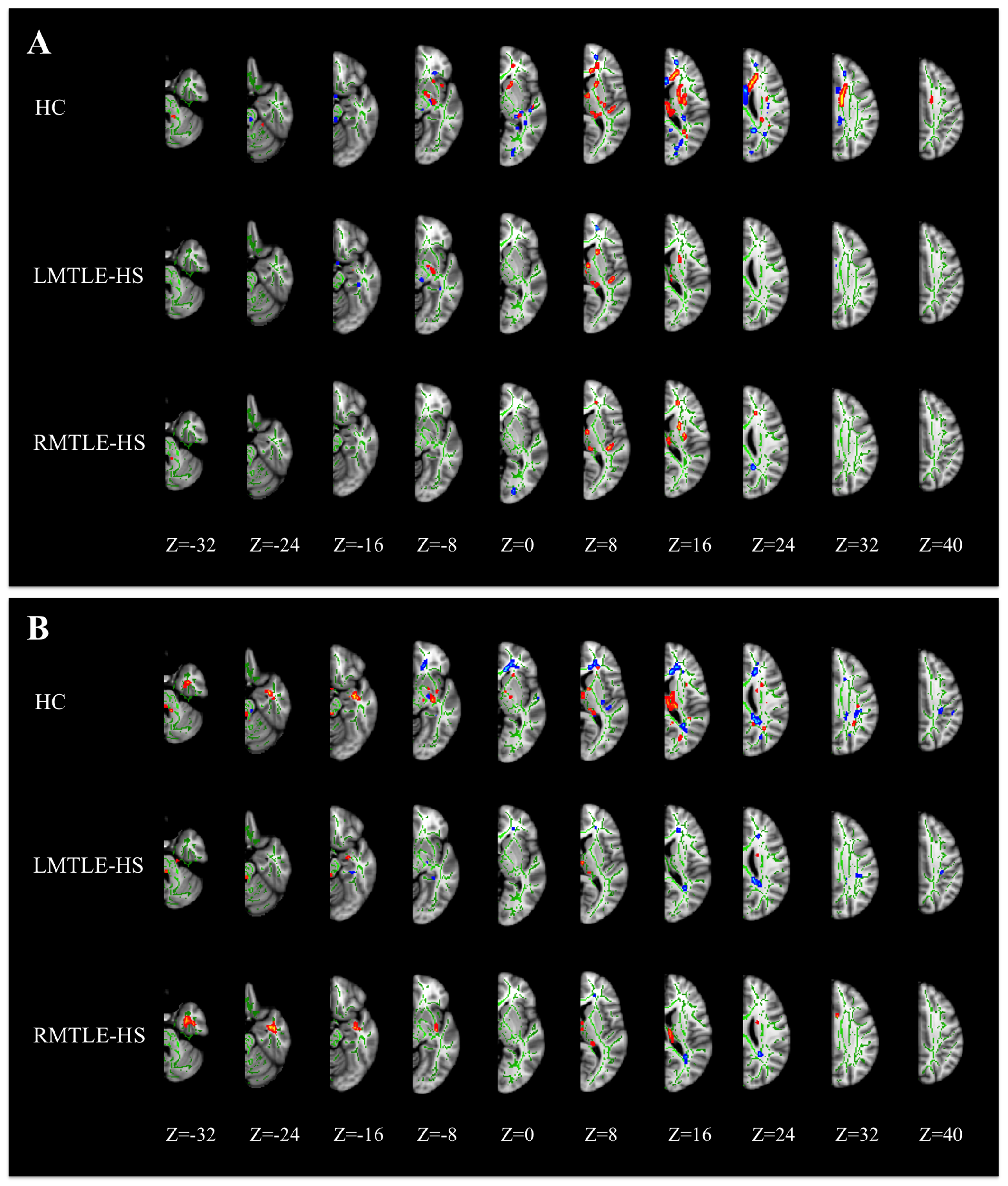


**Figure S1.** MRI images of significant tract-based spatial statistics (TBSS) clusters of white matter asymmetry in healthy controls, patients with left mesial temporal lobe epilepsy with hippocampal sclerosis (LMTLE-HS) and patients with right MTLE-HS. **(A)** represents axial diffusivity (AD) and **(B)** represents radial diffusivity (RD). Results are presented on the left hemisphere of the symmetric TBSS skeleton (depicted in green). Rightward asymmetry that was defined as having a larger AD/RD value of the right brain (R) than the left (L) (R>L) depicted in blue-light-blue and leftward asymmetry (L>R) in red-yellow.


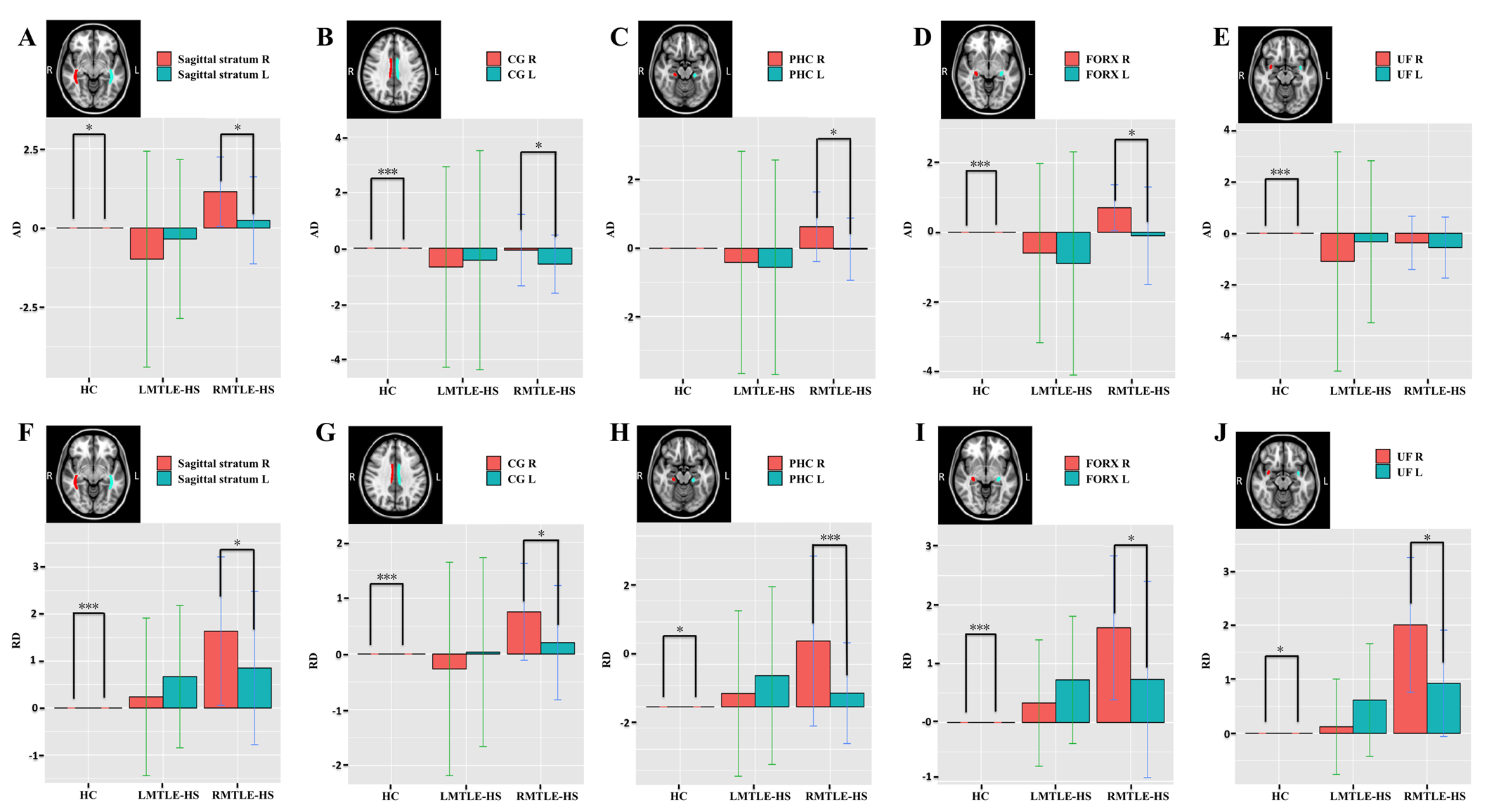


**Figure S2.** The paired-ROIs asymmetries in HC, LMTLE-HS and RMTLE-HS were calculated by comparing the AD (A-E) or RD (F-J) values of the paired-ROIs in bilateral hemispheres. The AD and RD values of LMTLE-HS and RMTLE-HS were calculated by using z scores based on the mean of the HC in a given hemisphere. Red represents the AD or RD values of the right hemisphere and blue represents the AD or RD values of the left hemisphere. Rightward asymmetry was defined as having a larger AD/RD value of the right brain than the left, and leftward asymmetry was left ROI value larger than right (**P*<0.05; ***P*<0.01; ****P*<0.001). CG, cingulum fibers within the cingulate gyrus; PHC, parahippocampal cingulum; FORX, fornix; UF, uncinate fasciculus.


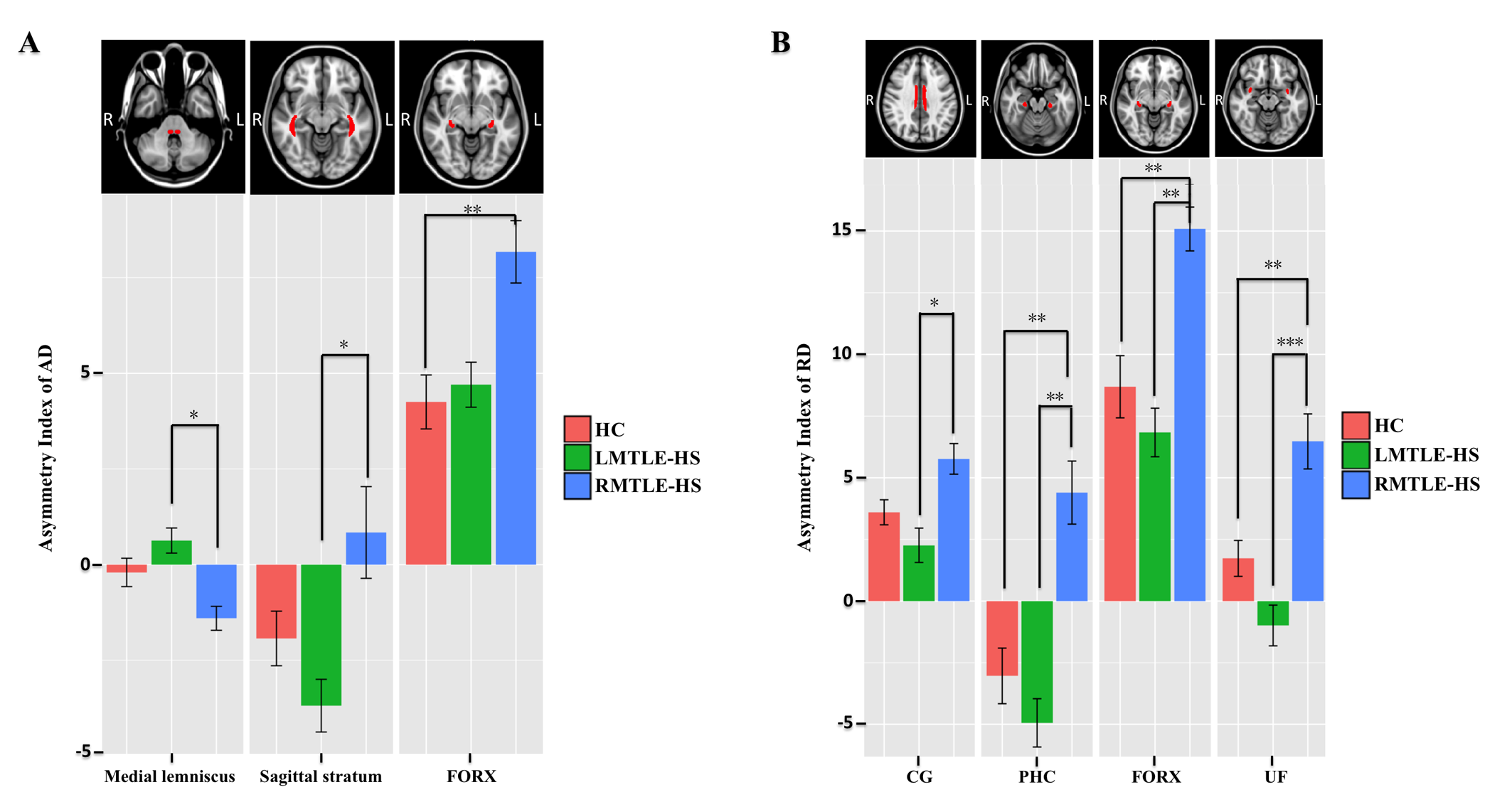


**Figure S3.** The asymmetry index (AI) differences of the paired-ROIs in bilateral hemispheres among HC, LMTLE-HS and RMTLE-HS. **(A)** represents the AI of AD and **(B)** represents the AI of RD. Red represents AD or RD values in HC; green, patients with LMTLE-HS; blue, patients with RMTLE-HS (**P*<0.05; ***P*<0.01; ****P*<0.001). FORX, fornix; CG, cingulum fibers within the cingulate gyrus; PHC, parahippocampal cingulum; UF, uncinate fasciculus.
